# Serum Oxidative Status and Thiol-Disulfide Homeostasis in Late-Gestation Holstein Heifers with and Without a History of Mid-Gestation Transport

**DOI:** 10.3390/vetsci13040356

**Published:** 2026-04-05

**Authors:** Güzin Özkurt, Recep Bozkurt, Tamer Kayar, Seynur Ali Hatib, Ayşenur Baydar

**Affiliations:** 1 Department of Biochemistry, Faculty of Veterinary Medicine, Aksaray University, Aksaray 68100, Türkiye; seynurhatib20@gmail.com (S.A.H.); aysenurbaydar18@gmail.com (A.B.); 2 Independent Researcher, Aksaray 68100, Türkiye; recep.435@hotmail.com; 3 Department of Animal Science, Faculty of Veterinary Medicine, Aksaray University, Aksaray 68100, Türkiye; tamerkayar@aksaray.edu.tr

**Keywords:** Holstein heifers, oxidative stress, pregnancy, transport stress

## Abstract

Pregnancy in dairy cattle is characterized by marked metabolic adaptations that may influence oxidative balance. In this study, oxidative stress markers and thiol–disulfide homeostasis were evaluated in transported and non-transported Holstein heifers during the last trimester of gestation. Clinically healthy 2-year-old heifers were divided into transported pregnant (*n* = 21) and non-transported pregnant (*n* = 9) groups. Blood samples were collected from the jugular vein approximately 90 days (3 months) after the mid-gestation transport event. These samples were analyzed for total antioxidant capacity (TAC), total oxidant status (TOS), oxidative stress index (OSI), malondialdehyde (MDA), native thiol (NTL), total thiol (TTL), and disulfide levels. Total oxidant status and oxidative stress index values were significantly higher in the non-transported group (*p* < 0.05). However, no significant differences were observed between groups in total antioxidant capacity, malondialdehyde, or thiol–disulfide parameters (*p* > 0.05). These findings suggest that metabolic adaptations specific to late gestation may influence systemic oxidant levels independently of transport exposure. Under the conditions of this study, transport did not induce a marked redox imbalance in pregnant Holstein heifers.

## Simple Summary

Pregnancy, particularly during the months approaching calving, is a period of intense metabolic activity in dairy heifers. During this time, the balance between oxidants and antioxidants may change as part of natural physiological adaptations. In dairy production systems, animals are sometimes transported internationally during pregnancy, and transportation can act as a stress factor. In this study, oxidative balance was evaluated during the last trimester of gestation in transported and non-transported Holstein heifers. Blood samples collected from both groups were analyzed for parameters related to oxidative status. The results showed higher oxidant levels in non-transported heifers, while no significant differences were observed between groups in antioxidant levels or thiol balance. These findings suggest that physiological adaptations specific to late pregnancy may contribute to oxidative changes independently of transport exposure. This highlights the importance of considering natural gestational changes when evaluating animal health and welfare in dairy cattle.

## 1. Introduction

Oxidative reactions constitute an essential component of cellular metabolism. During these reactions, the production of reactive oxygen species (ROS) may lead to metabolic disturbances when generated in excessive amounts [1]. ROS are intermediate products formed during metabolic processes. They are continuously produced in the body and, at low concentrations, play regulatory roles in various physiological functions [2]. Biologically generated ROS include superoxide radical (O_2_•^−^), hydrogen peroxide (H_2_O_2_), hydroxyl radical (•OH), and singlet oxygen (^1^O_2_). ROS are primarily produced in mitochondria under both physiological and pathological conditions [1,2,3].

The equilibrium of reactive oxygen species (ROS) is strictly governed by complex antioxidant defense mechanisms. Under normal physiological conditions, these systems provide sufficient capacity to neutralize pro-oxidant molecules. However, when ROS production overwhelms the cellular scavenging threshold, the resulting disruption of redox balance leads to oxidative stress, which can cause significant damage to vital macromolecules, including lipids, proteins, and DNA [4]. Specifically, lipid peroxidation results in the formation of cytotoxic end products such as malondialdehyde (MDA), while oxidative attacks on DNA can lead to base modifications and genomic instability. In this context, proteins are primary targets for both oxidative and nitrosative stress [5]. Reactive species can modify amino acid side chains, particularly the thiol (-SH) groups, leading to functional losses. Therefore, evaluating dynamic thiol–disulfide homeostasis is a crucial indicator, as it reflects the earliest stages of protein oxidation and systemic redox resilience [6,7].

To counteract excessive free radical production, the body utilizes both enzymatic and non-enzymatic antioxidant mechanisms [1,2]. The enzymatic antioxidant defense system includes superoxide dismutase (SOD), catalase (CAT), and glutathione peroxidase (GPx). Non-enzymatic antioxidants include glutathione, retinoids, carotenoids, ascorbic acid, vitamin E, and estrogens [2,8]. In addition, plasma albumin, an important non-enzymatic antioxidant component, binds free metal ions and thereby prevents free radical formation [9]. Excessive ROS production may cause peroxidative damage not only within cells but also in extracellular macromolecules. In farm animals, oxidative stress has been associated with reproductive disorders such as udder edema, retained fetal membranes, and mastitis [2].

Pregnancy and parturition are considered among the most physiologically vulnerable periods in dairy heifers [10,11]. During these stages, metabolic processes, including free radical generation, are markedly intensified [12]. It has been suggested that free radical production may be elevated during pregnancy relative to other physiological stages [3]. Consequently, pregnancy may represent a potential source of oxidative stress and imbalance between oxidant and antioxidant systems [13]. During gestation, metabolic reactions increase to meet the energy demands required for fetal development, colostrum synthesis, and the physical effort of parturition, potentially resulting in enhanced ROS production [14]. Elevated intrauterine ROS levels have been associated with complications such as intrauterine growth restriction, increased fetal mortality, and higher disease susceptibility. Moreover, strong relationships have been reported between maternal and fetal plasma antioxidant levels and oxidative stress status [15,16]. Prenatal oxidative stress may also influence postpartum physiological conditions [3].

The development of the fetal immune system begins during early gestation and continues to mature throughout pregnancy. Therefore, maternal metabolic and oxidative conditions during gestation may influence fetal development [15,17]. Exposure of pregnant heifers to oxidative stress during gestation may negatively affect the inflammatory responsiveness of calves [18]. In late gestation, high-yielding dairy heifers naturally transition into a state of negative energy balance (NEB), driven by the intensive metabolic demands of the rapidly growing fetus and significant hormonal shifts [11,19]. This physiological state is characterized by intense lipid mobilization and an accelerated production of ROS [20]. Consequently, these metabolic and oxidative challenges can predispose animals to severe clinical complications, including abortion, dystocia, and low birth weight in offspring [21].

Transport is widely recognized as one of the most significant stress factors in livestock production systems. During transportation, animals are exposed to multiple stressors including handling, loading procedures, environmental changes, feed and water deprivation, vibration, and social regrouping [22]. These stressors can activate the hypothalamic–pituitary–adrenal axis, leading to increased cortisol secretion and enhanced oxidative processes [23]. In pregnant animals, such stress exposure may further challenge redox homeostasis [24].

The methods developed by Erel for measuring total antioxidant capacity (TAC) and total oxidant status (TOS) are widely used in veterinary research. The oxidative stress index (OSI), calculated as the ratio of TOS to TAC, provides a comprehensive indicator of systemic redox balance [25,26]. Malondialdehyde (MDA), a lipid peroxidation product, is frequently used as a marker of membrane damage [27]. The oxidant–antioxidant balance has been associated with important physiological adaptations at different stages of pregnancy [28].

In addition, thiol–disulfide homeostasis has recently emerged as an important parameter in the evaluation of oxidative stress. Thiol groups (–SH) are functional protein groups that are sensitive to oxidative processes. Under oxidative conditions, thiol groups are converted into disulfide bonds (–S–S–), and this dynamic transformation is considered an important indicator of redox balance. Alterations in thiol–disulfide equilibrium may reflect systemic oxidative burden and cellular defense mechanisms [7]. These parameters serve as indicators of oxidative protein damage and reflect the level of nitrosative stress [29].

Although oxidative stress during pregnancy has been investigated in dairy cattle, data regarding the effects of transport stress on systemic oxidative markers and thiol–disulfide homeostasis in pregnant Holstein heifers remain limited. Therefore, the aim of the present study was to evaluate systemic oxidative status in transported pregnant Holstein heifers and to compare TAC, TOS, OSI, MDA, and thiol–disulfide parameters with those of non-transported pregnant control heifers.

In recent years, Türkiye has imported a significant number of high-yielding cattle breeds for both breeding and production purposes to meet increasing domestic demand. A substantial portion of these imports consists of pregnant heifers, primarily sourced from European countries. These animals are often subjected to long-distance international transport, which involves various physical and psychological stressors, including fluctuating temperatures, noise, vibrations, and social disruption. While the immediate effects of transport stress are well-documented, the long-term physiological consequences, especially regarding the oxidative status and acclimatization of pregnant heifers, remain a critical area of concern for animal welfare and productivity [30].

One of the most significant challenges in the livestock sector is the high incidence of reproductive failures, particularly late-term abortions, in heifers imported from Europe. While infectious diseases are frequently monitored, the role of metabolic exhaustion and oxidative damage caused by long-distance transport remains largely unaddressed. Oxidative stress is known to adversely affect placental function and fetal development in cattle; however, data regarding the chronic effects of transport on systemic oxidative markers and thiol–disulfide homeostasis in pregnant Holstein heifers remain limited. The objective of this study was to evaluate the long-term impact of long-distance transport (3800 km) during mid-gestation on the MDA, TOS, TAC, and the resulting OSI, alongside thiol–disulfide homeostasis in pregnant heifers. We hypothesized that long-distance transport during mid-gestation (5th month) might induce a persistent ‘oxidative footprint’, characterized by a disruption in the balance between TOS and TAC, which would remain detectable during late gestation (8th month). We propose that this chronic oxidative imbalance, reflected by an increased OSI, altered thiol–disulfide homeostasis, and elevated lipid peroxidation (MDA), may serve as a predisposing factor for the increased vulnerability of imported heifers to late-term complications, such as abortions, by compromising the maternal antioxidant defense system and overall physiological resilience.

## 2. Materials and Methods

### 2.1. Animals and Study Design

This study was conducted on 30 clinically healthy, 2-year-old Holstein heifers in their last trimester of gestation (8th month). Based on clinical examinations and farm health records, the animals were assigned to two groups: transported pregnant (TG; *n* = 21) and non-transported pregnant (NG; *n* = 9). The TG consisted of nulliparous heifers imported from Denmark to Türkiye, covering a distance of approximately 3800 km. To ensure uniform exposure to transport-related stressors, all heifers in the TG were transported together in a single shipment using the same vehicle, following an identical transport protocol and route. The animals were moved during the 5th month of their first pregnancy. Following arrival, all heifers underwent a 4-month acclimatization period in the same facility under identical environmental and nutritional conditions as the NG group until sampling in late gestation.

The animals were fed a Total Mixed Ration (TMR) formulated to meet their nutritional requirements during late gestation. The daily ration for the pregnant heifers consisted of straw (3.7 kg), barley silage (4.5 kg), corn silage (6.7 kg), corn flakes (1.8 kg), and alfalfa hay (0.6 kg). Additionally, each animal received 1.8 kg of concentrate feed, supplemented with a vitamin-mineral premix (0.1 kg) and a toxin binder (0.015 kg). The concentrate was formulated to provide high-quality protein. Fresh water was provided ad libitum. All subjects were housed in semi-open, free-stall barns with natural ventilation on a commercial farm in Aksaray, Türkiye (38°19′11.7″ N, 33°54′13.7″ E)

### 2.2. Blood Sampling and Serum Preparation

Blood samples were collected in the 8th month of gestation, approximately 90 days after the transport event occurred in the 5th month. Blood samples were collected from the vena jugularis into serum tubes (Vacusera, Disera, İzmir, Türkiye) under standard aseptic conditions. The samples were centrifuged at 3000 rpm for 10 min (NF 800, Nüve, Ankara, Türkiye), and the obtained serum was stored at −20 °C (Uğur Soğutma, Aydın, Türkiye) until biochemical analyses were performed.

### 2.3. Determination of Thiol/Disulfide Homeostasis

Serum thiol–disulfide homeostasis was determined using the automated spectrophotometric method described by Erel and Neselioglu. In the first step, dynamic and reducible disulfide bonds were reduced to free functional thiol groups, while structural (static) disulfide bonds remained unaffected. Subsequently, excess reducing agent (NaBH_4_; sodium borohydride) was neutralized. In the final step, total thiol (TTL) groups, comprising both native thiols (NTL) and reduced disulfide bonds, were reacted with DTNB (5,5′-dithiobis-(2-nitrobenzoic acid), Ellman’s reagent). The colorimetric measurements were performed at a wavelength of 415 nm using an automated clinical chemistry analyzer. All thiol parameters were expressed in µmol/L [7]. All biochemical measurements were performed using a fully automated biochemistry analyzer (BS-300, Mindray Medical International Ltd., Shenzhen, China) with commercial kits (Otto Scientific, Ankara, Türkiye).

Disulfide levels were calculated as half of the difference between total thiol and native thiol concentrations. After determining native thiol (−SH), disulfide (−S−S−), and total thiol [−SH + (−S−S−)] levels, the disulfide/native thiol (%) and disulfide/total thiol (%) ratios were calculated.

### 2.4. Measurement of Total Antioxidant Capacity (TAC)

Total antioxidant capacity (TAC) was measured using the method developed by Erel. Reagent 1 was prepared in Clark buffer at pH 1.8 and contained 10 mM o-dianisidine diluted at a ratio of 1:75 and 45 mM ammonium ferrous sulfate. Reagent 2 was prepared using Clark buffer at pH 1.8 and contained 7.5 mM hydrogen peroxide (H_2_O_2_). During the assay, the Fe^2+^–o-dianisidine complex present in Reagent 1 reacted with hydrogen peroxide in Reagent 2 through a Fenton-type reaction, generating hydroxyl radicals. These hydroxyl radicals reacted with o-dianisidine, resulting in color formation. Antioxidants present in the samples suppressed this oxidative reaction, thereby limiting color development. After completion of the reaction, absorbance values were measured spectrophotometrically at 660 nm and TAC levels were calculated accordingly. Total antioxidant capacity (TAC) was determined using an automated colorimetric method, and the results were expressed in mmol Trolox equiv./L [25].

### 2.5. Determination of Total Oxidant Status (TOS)

Total oxidant status (TOS) was determined using the colorimetric method developed by Erel. Reagent 1 was prepared based on a stock solution containing 25 mM sulfuric acid (H_2_SO_4_) dissolved in 140 mM NaCl. To this solution, glycerol (10%) and 250 µM xylenol orange were added sequentially. Reagent 2 was prepared using the same stock solution, followed by the addition of 10 mM o-dianisidine dihydrochloride and 5 mM ammonium ferrous sulfate. During the assay, oxidants present in the samples oxidized the ferrous ion–o-dianisidine complex to ferric ions. Glycerol in the reaction medium enhanced the oxidation process. The resulting ferric ions formed a colored complex with xylenol orange under acidic conditions. The intensity of the color was directly proportional to the total oxidant level of the sample. Absorbance values were measured at 546 nm using the end-point method. Hydrogen peroxide (H_2_O_2_) was used for calibration, and results were expressed as µmol H_2_O_2_ equivalent/L [25].

### 2.6. Calculation of Oxidative Stress Index (OSI)

Oxidative stress index (OSI), an indicator of oxidative stress, was calculated as the ratio of total oxidant status (TOS) to total antioxidant capacity (TAC). OSI values were expressed as arbitrary units (AU) [31,32,33] according to the following formula:

OSI (Arbitrary Unit) = TOS (µmol H_2_O_2_ equivalent/L)/TAC (µmol Trolox equivalent/L)

### 2.7. Malondialdehyde (MDA) Determination

Serum malondialdehyde (MDA) levels were determined using the thiobarbituric acid (TBA) reaction method. This assay is based on the formation of a pink chromogen resulting from the reaction between MDA (or MDA-like substances) and TBA under high temperature and acidic conditions.

Briefly, serum samples were mixed with two volumes of cold 10% (*w*/*v*) trichloroacetic acid (TCA) to precipitate proteins. After centrifugation, an aliquot of the supernatant was reacted with an equal volume of 0.67% (*w*/*v*) TBA solution. The reaction mixture was incubated in a boiling water bath (90–100 °C) for 10 min under acidic conditions (pH 2–3). Following cooling to room temperature, absorbance was measured spectrophotometrically at 532 nm. MDA levels were calculated based on the absorbance values obtained. MDA concentrations were measured as an indicator of lipid peroxidation, and the results were expressed in µmol/L [34].

### 2.8. Statistical Analysis

Statistical evaluation of the data was performed using IBM SPSS Statistics software (version 27.0). Normality of data distribution was assessed using the Shapiro–Wilk test. Comparisons between groups were performed using the independent samples *t*-test for normally distributed variables and the Mann–Whitney U test for non-normally distributed variables. Data are presented as mean ± standard deviation (SD) for normally distributed variables and median (interquartile range, IQR) for non-normally distributed variables. A *p*-value < 0.05 was considered statistically significant.

## 3. Results

### 3.1. Oxidative Stress Parameters

The oxidative stress parameters of transported pregnant (TG) and non-transported pregnant (NG) Holstein heifers are presented in Table 1.

Statistical analysis revealed that TOS and the OSI were significantly lower in the transported group compared to the control group (*p* = 0.002 and *p* = 0.013, respectively). In contrast, TAC levels remained stable and showed no significant difference between the two groups (*p* = 0.982). Regarding lipid peroxidation, MDA levels exhibited a numerical increase in the TG; however, this trend did not reach the conventional threshold of 0.05, although it notably approached statistical significance (*p* = 0.067).

### 3.2. Thiol–Disulfide Homeostasis

Thiol–disulfide parameters are summarized in Table 1.

No statistically significant differences were observed between the groups in terms of total thiol (TTL), native thiol (NTL), and disulfide levels (*p* > 0.05). However, native thiol levels in the TG exhibited a decreasing trend approaching statistical significance compared to the NG (*p* = 0.066). All other thiol–disulfide homeostasis parameters remained similar between the two groups.

## 4. Discussion

In this study, oxidative stress markers and thiol–disulfide homeostasis were evaluated during late gestation (8th month) in clinically healthy Holstein heifers that had been transported during mid-gestation (5th month), representing a sampling interval of approximately 90 days (3 months) post-transport, compared to a non-transported (NG) control group. The main findings demonstrated that total oxidant status (TOS) and oxidative stress index (OSI) levels were significantly higher in the NG group, whereas no significant differences were observed between groups in terms of total antioxidant capacity (TAC), malondialdehyde (MDA), or thiol–disulfide parameters.

The last trimester of pregnancy is characterized by rapid fetal growth, increased metabolic rate, and elevated oxygen consumption. During this period, enhanced mitochondrial respiratory activity may physiologically increase reactive oxygen species (ROS) production [20,35]. Our results align with the comprehensive review by Tufarelli et al. [35], which emphasizes that physiological stages such as late pregnancy and peak lactation are characterized by significant metabolic adjustments that inherently escalate oxidative stress. The elevated TOS and OSI levels in the NG group suggest that metabolic disruptions, primarily driven by the transition toward parturition and the resulting negative energy balance (NEB), contribute to increased ROS production independently of external stressors. In late gestation, high-yielding dairy heifers naturally enter a state of negative energy balance (NEB) due to the increased metabolic demands of the rapidly growing fetus and hormonal shifts. This period is characterized by intense fat mobilization and increased production of reactive oxygen species (ROS). Although long-distance transport was hypothesized to exacerbate metabolic strain and deplete antioxidant reserves, our data did not demonstrate a persistent oxidative shift in the transported group under the conditions of this study.

Furthermore, Sharma et al. [36] observed elevated MDA and altered antioxidant enzyme activities (SOD and GPx) during advanced gestation, marking it as a period of high lipid peroxidation. Similarly, Konvičná et al. [37] reported that oxidative pressure peaks during the transition period, with MDA concentrations being significantly higher immediately after calving compared to late pregnancy. While these researchers focused on the acute transition to calving, our data show that oxidative stress is already prominent in the 8th month of gestation.

Transport is an important stress factor associated with activation of the hypothalamic–pituitary–adrenal axis and increased cortisol secretion. However, its impact on oxidative responses may vary depending on duration, intensity, environmental conditions, and the timing of blood sampling [22]. The absence of increased oxidant markers in the TG group in this study contradicts traditional models of acute transport stress, such as those described by Minka and Ayo [23], which typically report elevated oxidative markers immediately following transit. This result may be attributed to the timing of sampling, which was conducted approximately 90 days post-transport, potentially suggesting a capacity for metabolic adaptation in the heifers. It is possible that the earlier transport event triggered a hormetic effect, potentially “priming” the antioxidant defenses for the subsequent demands of late gestation, as opposed to the un-primed oxidative surge seen in the NG group.

The lack of significant differences in TAC levels between groups indicates that systemic antioxidant capacity may have been preserved. Although MDA levels tended to be higher in the TG group, this difference was not statistically significant. Previous literature suggests that lipid peroxidation markers typically show marked increases under prolonged or severe oxidative stress conditions [4]. Unlike the significant changes in MDA observed by Sharma et al. and Konvičná et al. [36,37] during the acute stress of calving, the heifers in our study maintained functional redox resilience. In addition, a notable inter-individual variability was observed in MDA levels. However, this variability is unlikely to be attributed to transport-related stress, as similar interquartile range distributions were evident in both NG and TG groups. This consistent dispersion pattern across groups suggests that the variability primarily reflects inherent biological differences, such as individual metabolic status or oxidative stress responsiveness, rather than a direct consequence of transport.

Thiol–disulfide homeostasis represents an important indicator of protein-based redox regulation and reflects the organism’s antioxidant buffering capacity. Recent studies have demonstrated that thiol balance is sensitive to metabolic stress and oxidative alterations [7]. However, no significant differences were detected in native thiol, total thiol, or disulfide levels in the present study. Using the sensitive Erel and Neselioğlu method [7], our results suggest that the heifers’ protein-based antioxidant reservoirs remained functionally resilient and capable of buffering oxidative challenges, keeping oxidative pressure at a subclinical level, as discussed by Tufarelli et al. [35].

Several limitations should be considered when interpreting these findings. Oxidative markers may be influenced by gestational stage, nutritional status, energy balance, and antioxidant micronutrient levels [4,38]. Additionally, the relatively small sample size of the NG group may have limited the statistical detection of subtle differences in certain parameters.

From a broader perspective, understanding redox adaptations during late pregnancy and the impact of management-related stressors is important for improving welfare and herd management strategies in dairy cattle. Future studies including larger sample sizes, clearly defined gestational stages, controlled transport durations, and longitudinal sampling designs are recommended. Furthermore, evaluation of inflammatory and endocrine markers may provide deeper insight into the relationship between metabolic stress and systemic redox regulation.

## 5. Conclusions

This study evaluated oxidative stress markers and thiol–disulfide homeostasis during the late gestation period (8th month) in pregnant Holstein heifers that had been transported during mid-gestation (5th month), compared to a non-transported control group. Our findings demonstrated significantly higher TOS and OSI levels in the non-transported heifers (NG), whereas TAC, MDA, and thiol–disulfide parameters did not exhibit significant differences between groups. These results suggest that late-gestation metabolic adaptations, potentially associated with negative energy balance (NEB), appear to be significant factors influencing systemic oxidant status. Under the conditions of this study, these metabolic changes may have a more relevant influence than the history of mid-gestation transport, which did not result in a detectable long-term oxidative impact.

In conclusion, no evidence of increased long-term oxidative burden associated with previous transport was observed at late gestation under the conditions of this study. While the relatively small and unbalanced sample size (*n* = 9 vs. *n* = 21) represents a limitation, the use of a single breed (Holstein-Friesian) at an identical gestational stage helped minimize genetic and physiological variability. These findings suggest that physiological gestational shifts should be considered when evaluating oxidative dynamics in dairy cattle. Future longitudinal research with larger, balanced populations and sampling immediately before and after transport is warranted to further clarify the interaction between metabolic adaptation and oxidative regulation in imported pregnant heifers.

## Figures and Tables

**Table 1:** Comparison of oxidative stress and thiol–disulfide parameters between non-transported pregnant (NG) and transported pregnant (TG) Holstein heifers.

Parameter	NG (*n* = 9)	TG (*n* = 21)	*p*-Value
Total thiol (µmol/L)	306.00 (170.00)	237.00 (99.00)	0.167
Native thiol (µmol/L)	193.00 ± 80.83	142.95 ± 58.42	0.066
Disulfide (µmol/L)	50.50 (51.00)	46.00 (59.50)	0.734
TAC (mmol Trolox Eq/L)	1.26 (0.61)	1.32 (0.26)	0.982
TOS (µmol H_2_O_2_ Eq/L)	8.44 ± 1.45	5.57 ± 2.36	0.002 *
OSI (AU)	0.620 ± 0.170	0.425 ± 0.190	0.013 *
MDA (nmol/mL)	5.15 (1.51)	6.12 (1.75)	0.067

Data are presented as mean ± standard deviation (SD) for normally distributed variables and median (interquartile range, IQR) for non-normally distributed variables. Comparisons were performed using the independent samples *t*-test or Mann–Whitney U test, as appropriate. (* *p* < 0.05 indicates statistical significance).

## Data Availability

The original contributions presented in the study are included in the article, further inquiries can be directed to the corresponding author.

## Abbreviations

The following abbreviations are used in this manuscript:

MDA	Malondialdehyde
NTL	Native Thiol
NEB	Negative Energy Balance
NG	Non-Transported Pregnant
OSI	Oxidative Stress Index
ROS	Reactive Oxygen Species
TAC	Total Antioxidant Capacity
TOS	Total Oxidant Status
TTL	Total Thiol
TG	Transported Pregnant
